# Assessment of Fast-Growing and Dual-Purpose Chicken Meat Quality Characteristics in Different Production Systems

**DOI:** 10.3390/ani16020272

**Published:** 2026-01-16

**Authors:** Ioannis-Emmanouil Stavropoulos, Georgios Manessis, Zoitsa Basdagianni, Aikaterini Tsiftsi, Anne-Jo Smits, Peter van de Beek, Vasilios Tsiouris, Georgios Arsenos, Ioannis Bossis

**Affiliations:** 1 Laboratory of Animal Husbandry, Department of Animal Production, School of Agriculture, Aristotle University of Thessaloniki, 54124 Thessaloniki, Greece; istavrop@agro.auth.gr (I.-E.S.); gmanes@agro.auth.gr (G.M.); basdagianni@agro.auth.gr (Z.B.); atsifts@agro.auth.gr (A.T.); 2 Poultry and Calf Expertise Centre, Aeres University of Applied Sciences, 6717 Barneveld, The Netherlands; a.smits@aeres.nl (A.-J.S.); p.vande.beek@aeres.nl (P.v.d.B.); 3 Unit of Avian Medicine, Clinic of Farm Animals, School of Veterinary Medicine, Aristotle University of Thessaloniki, 54124 Thessaloniki, Greece; biltsiou@vet.auth.gr; 4 Laboratory of Animal Production & Environmental Protection, School of Veterinary Medicine, Faculty of Health Sciences, Aristotle University of Thessaloniki, 54124 Thessaloniki, Greece; arsenosg@vet.auth.gr

**Keywords:** poultry, intrinsic quality, slaughter age, seasonal effect, fatty acid profile

## Abstract

This study focused on comparing broiler meat quality across different production systems and seasons. Chicken carcasses from intensive, free-range, and dual-purpose poultry systems were analyzed for intrinsic and extrinsic quality traits. The results revealed significant effects of the production system and season. Carcasses from dual-purpose and intensive systems were heavier. Greater carcass weight was recorded in autumn and winter. The mean post-mortem pH of breast and thigh was lower in extensive and dual-purpose systems and significantly lower in winter and spring. Colorimetric parameters varied by system, as higher means of redness (intensive), yellowness (free-range), and lightness (dual-purpose) were observed. Meat from intensive systems was less firm, showed higher levels of unsaturated fatty acids and better oxidation stability. Dual-purpose displayed higher levels of polyunsaturated fatty acids. The interaction effect was significant for most quality parameters.

## Simple Summary

Poultry meat is an important source of high-quality protein in healthy diets, and its demand continues to rise. Consumer preferences increasingly influence this demand, driven by concerns about chicken welfare and its potential effect on meat quality. These concerns have strengthened the perception that non-intensive production systems yield higher-quality poultry products. Meat quality, however, is shaped by multiple factors, including flock management, rearing conditions, feeding practices, and handling procedures, as well as seasonal variations that affect indoor and outdoor environments. This study assessed quality traits of poultry meat from the following three distinct production systems: intensive, extensive, and dual-purpose across four seasons. Meat from intensive systems was tender, had a higher content of unsaturated fatty acids, and showed better oxidation stability. Extensive systems produced lighter carcasses and displayed lower final pH in the breast and thigh. Meat from dual-purpose systems had a lighter color, a higher moisture content, higher levels of polyunsaturated fatty acids, and lower collagen content. The results also varied by season. The findings contrasted with prevailing consumer perceptions of meat quality in alternative production systems; thus, it is suggested that informed consumer choices require clear, scientifically backed information.

## 1. Introduction

The global demand for poultry meat continues to grow, driven by its affordability, nutritional value, and culinary versatility [1]. This rising demand underscores the importance of evaluating broiler meat quality, which is a complex interplay of quantifiable traits and sensory characteristics. Scientifically, meat quality is assessed through parameters such as visual appeal (color consistency), texture (firmness), pH, nutrient content and shelf life [2,3]. However, consumer expectations often transcend these technical measures. Consequently, buyers base their decisions on attributes they prioritize most, such as freshness, perceived health benefits, palatability, and firmness. [3]. Additionally, the growing awareness of sustainability and animal welfare issues, which are often linked to the intensification of animal production, has shifted the demand towards products originating from alternative farming systems [4,5]. This has recently led to the introduction of dual-purpose poultry-production systems to address sustainability and welfare issues [6].

Production or farming systems are categorized in relation to the type and quantity of inputs used for production purposes [7]. High-input systems, which dominate global production, rely on fast-growing breeds, nutrient-dense feeds, and controlled housing to maximize efficiency [8,9]. Conversely, low-input systems, such as extensive systems (ESs) or dual-purpose systems (DPSs), prioritize slower growth, implement a diet based on natural forages, and allow access to outdoor spaces in lower flock stocking densities [8]. Growth rate and stocking density are both management practices with welfare and financial importance [10]. Considering the above, it is evident that meat quality and the production system (PS) are interlinked. Meat quality is also affected by seasonal effects, as weather or climate conditions influence the biochemical conditions of muscles before slaughter [3]. Lately, consumer attitudes have shown a preference for meat from low-input PSs, associating them to higher standards of welfare and product quality, reflecting a rising portion in the market of these systems [11,12]. Despite these distinctions, comprehensive studies comparing the impacts of PS and season on objective quality metrics are limited, especially for the DPSs. This study focused on evaluating poultry meat quality across high- and low-input farming systems, integrating scientific measurements of key quality parameters with insights into consumer preferences. Through the examination of both external and internal meat traits, this research aims to provide a comprehensive perspective on how PS and season influence meat quality. The findings are intended to guide sustainable poultry farming practices that meet industry standards while also addressing consumers’ evolving expectations.

## 2. Materials and Methods

### 2.1. Production Systems and Samplings

The study was conducted over a two-year period (2022–2024) and included a total of six broiler farms. Of these, two operated under an intensive production system (IS), two under an extensive production system (ES), and two under a dual-purpose production system (DPS). Specifically, ISs use commercial fast-growing lines (Ross 308), whereas ESs and DPSs use slow-growing lines (Hubbard in ESs and Sasso silver in DPSs). In ISs, up to 65,000 broilers were housed at a stocking density of 39 Kg/m^2^. In ISs, birds were reared in closed poultry houses with mechanical ventilation and controlled indoor microclimate, according to standard commercial broiler management practices. ESs represented moderate-sized facilities with outdoor access (35,000 housed broilers at a stocking density of 19–22 Kg/m^2^. These characteristics are displayed in Table 1. In contrast, ESs and DPSs were based on open or semi-open housing with outdoor access, where ventilation and environmental exposure followed natural conditions. In DPSs birds also had outdoor access and were kept at a density of 12 Kg/m^2^. The chemical composition of feed of IS broilers is summarized in Table 2. The main ingredients included toasted soybeans, soybean oil, maize, wheat, toasted palm kernel, rapeseed oil, lecithin, and palm oil. The diet of ESs broilers was mainly based on forage, predominantly grasses, while a mixture of pellets with the addition of lucerne and straw was given for indoor foraging when outdoor access could not be provided. In DPS, broilers had daily access to outdoor pastures for grazing and were offered a pellet feed as supplement consisting of maize, wheat, sunflower oil, oat and minerals (Table 3). Processing was carried out at commercial slaughterhouses associated with each farm, following comparable standard industrial procedures for transport, lairage, stunning, slaughter, and carcass chilling. Deboning and sample collection were performed at similar post-mortem times across all systems and seasons. After collection, carcasses were vacuum-packed, stored at 4 °C, and laboratory analyses were initiated within 24 h post-slaughter. Sampling followed a fully factorial farm × season × production system design. Specifically, two farms were sampled per production system (IS, ES and DPS), and each farm was sampled once per season (winter, spring, summer and autumn). This resulted in 24 independent sampling events (4 seasons × 3 production systems × 2 farms). Whole carcasses were collected from slaughterhouses that processed the broilers from the pilot farms. Slaughter age depended on the production system; broilers from intensive systems were slaughtered at 38–42 days of age, while chickens from extensive and dual-purpose systems were slaughtered at 56–70 and 100 days, respectively, to reflect slower growth rates. At each farm × season × system sampling, a subset of 10 carcasses was collected. A total of 230 breast and 230 thigh samples were included in the final dataset, as one seasonal sampling could not be fully retained due to sample condition at receipt. All breast meat analyses were performed on the *Pectoralis major* (PM) muscle, while all thigh meat analyses were performed on the *Tibialis anterior* (TA) muscle. The gross chemical composition, fatty acid profile, pH (24 h post-mortem), meat color, firmness, and oxidative stability were analyzed in PM samples. TA samples were used for pH (24 h post-mortem) and fatty acid profile analyses only.

### 2.2. Meat Physicochemical Properties

Upon arrival at the laboratory, the carcasses were weighed to measure carcass weight (CW) in grams (Scaltec Instruments GmbH., Göttingen, Germany). Βreast (PM) and thigh (TA) pH measurements were recorded with a pH meter (Hanna instruments, Woonsocket, RI, USA), equipped with a glass electrode (FC2023). The electrode was calibrated by using standard pH buffer solutions (pH values of 4.00 and 7.00). For chicken breast color, parameters were determined with a colorimeter (CR-310, Minolta, Tokyo, Japan), based on the CIELAB system [13]. Color measurements were taken at three random locations of each sample. The parameters recorded were lightness (L*, ranging from 0 for black to 100 for white), redness (a*, ranging from −60 for green to +60 for red) and yellowness (b*, ranging from −60 for blue to +60 for yellow), which were additionally used for the calculation of chroma (Ch) and hue angle (Hue) values [14].

The evaluation of firmness (F) was performed in raw meat parts from the PM muscle (1.3 cm diameter, 3–4 cm length). The muscle fibers in the meat parts were oriented parallel to the core to ensure perpendicular cuts. A texture analyzer (Instron Corporation, Norwood, MA, USA) was used to record the shear force needed (N) to cut the sample. A Warner–Bratzler V-shaped blade (1.02 mm thickness, 60° angle) was used for the cutting of samples.

The gross chemical composition of breast meat (% fat, protein, moisture, collagen) was analyzed using the DA 7250 At-line Near-Infrared (NIR) Instrument (Perten Instruments, Hagersten, Sweden), with the calibrations being pre-installed by the manufacturer, following the standard procedures [15] (. The meat samples were minced before the analysis.

### 2.3. Meat Fatty Acid Profile Analysis

Fatty acid methyl esters were prepared for gas chromatography (GC) analysis with a modification of the protocol introduced by O’Fallon et al. (2007) [16]. In detail, 1 g of sample was placed in a screwcap Pyrex tube and was hydrolyzed with potassium hydroxide-water solution (0.7 mL) and then methanol-BHT solution (5.3 mL) was added. The mixture was placed in a water bath at 54 °C for 90 min. Afterwards, 0.58 mL of aqueous sulfuric acid (24 N) was added, followed by an incubation step at 55 °C for 90 min in a water bath. Tubes were cooled at room temperature and 3 mL of hexane (95%) was added. Tubes were centrifuged at 2500× *g* for 2 min and the supernatant layer was collected and analyzed via gas chromatography, with an Agilent chromatograph 6890 N, equipped with a flame ionization detector and a capillary column DB-23, 60 m-0.25 mm i.d., 0.25 μm film thickness (Agilent Technologies, Santa Clara, CA, USA). The amounts of individual fatty acids were determined by measuring their corresponding peak areas, and the results were reported as percentages (%) relative to the sum of the peak areas of all identified acids.

### 2.4. Meat Oxidation Stability

The samples were collected and placed in the refrigerator (4 °C) for three days, to simulate a three-day storage time. TBARS (Thiobarbituric acid reacting substances) analysis was performed as follows:

Ten grams of sample were weighed into a conical Erlenmeyer flask and 1 mL of 1% BHT in pure ethanol (98.9%) was added to prevent autoxidation of PUFAs. A total of 35 mL of perchloric acid (3.86%) was added to the flask and the mixture was homogenized with a Wisd homogenizer (HG-15D, Daihan Scientific, Seoul, South Korea) until the complete dissolution of the sample. Filtration was performed with a filter paper (F1002 grade, 110 mm, CHMLAB Group, Barcelona, Spain) and 5 mL of clear sample solution was transferred to a 15 mL glass tube. Five mL of aqueous Thiobarbituric acid 0.02 Μ (TBA) was added and the tubes were vortexed and then put into the water bath for 35 min at 99 °C. Subsequently, the tubes were removed and placed at room temperature to cool down for 15 min and cooled with tap water. The absorbance of the solution recorded with a UV–VIS double beam spectrophotometer at 532 nm (Halo DB-20S, Dynamica Scientific Ltd., Livingston, UK) to measure the malondialdehyde content (MDA). The TBARS were calculated using 1,1,3,3 tetraethoxypropane as standard and the result was read as mg of MDA per Kg. The samples were analyzed in duplicates, and the mean value was used. For the blank solution, 5 mL of deionized water were mixed with 5 mL TBA.

### 2.5. Statistical Analysis

Data for all examined traits were analyzed using Mixed Linear Models within an Analysis of Variance (ANOVA) framework [17]. All models included fixed effects (main and interaction) for the factors “Production System” (PS: IS, ES, DPS) and “Season” (S: autumn, winter, spring, summer). Because the same farms were sampled across seasons, season was specified as a repeated factor at the farm level. Accordingly, Farm (six levels) was included as a random subject effect, nested within Production System. Differences among treatments’ mean values were tested using the Least Significant Difference (LSD) test. The normality and the homoscedasticity of the models’ residuals were assessed, and no substantial violations were detected. In all hypothesis testing procedures, the significance level was predetermined at *a* = 0.05 (*p* ≤ 0.05). All statistical analyses were carried out using IBM SPSS Statistics (v.29).

## 3. Results

### 3.1. Physicochemical Properties

The results from the analysis of physicochemical properties (Table 4) showed that CW differed significantly among PSs, with carcasses from the DPSs being the heaviest (1828 ± 35.6 g), followed by those from the ISs (and 1750 ± 32.0 g). The seasonal effect was also significant as carcasses from the autumn and winter samplings displayed higher means [(1870 ± 36.0 g) and (1827 ± 35.4 g), respectively]. The interaction effect (S × PS) was also significant and showed that bigger CW was recorded in ESs and DPSs for autumn and winter, while the CW from ISs was the heaviest recorded in summer. The mean pH value in PM varied significantly between PS, with the lowest mean observed for ES (5.66 ± 0.02). The mean pH in TA showed a similar trend; samples from IS exhibited the highest mean while winter and spring samplings displayed the lowest. A significant effect of season was also observed, winter and spring samplings showing lower means (5.67 ± 0.02). During autumn and winter, the IS presented significantly higher means for both PM and TA. In contrast, the mean pH from ES was the lowest in spring (PM), and summer (TA). The DPS had the lowest mean pH (TA) for autumn and winter.

Regarding the colorimetric parameters, DPSs carcasses showed a significantly higher mean of L* (64.8 ± 0.34) and a lower mean of a* (11.0 ± 0.20), whereas ES carcasses showed greater b* values (17.6 ± 0.20). The seasonal effect was significant for the mean L (higher in summer) and b* (higher in autumn) values. A significant interaction effect was detected showing that the mean L* value was also higher in DPS (winter, summer), but the mean a value was the lowest (autumn, summer). The mean Ch and Hue differed significantly across PSs, with the highest means observed in ESs. These traits presented higher means in the autumn. According to the S × PS analysis (Table S1), these values were also greater for ESs within all seasons.

Firmness (F) was also affected by the PS. Meat cuts from PM muscle from DPSs displayed higher mean values of firmness (102.6 ± 2.60 N) while those from ISs presented the lowest values (82.4 ± 2.30 N). The seasonal effect was not significant; however, there was a significant interaction effect, indicating that carcasses from DPS and ES displayed greater means (autumn and summer).

Fat and collagen contents (Table 3) were significantly higher for the ISs (1.42 ± 0.03 and 0.51 ± 0.01%, respectively), while the percentage of protein and moisture was found to be higher for the DPSs (75.9 ± 0.06%). Significant seasonal variations were observed in fat content, with a lower mean in autumn, whereas protein content showed a slightly lower mean in spring. Regarding the interaction effect, fat and collagen contents were found to be significantly elevated in ISs within seasons. Similarly, the protein and moisture content exhibited the same trend for DPSs within seasons, except the summer season, in which the mean was not significantly different to that of the ESs (Table S1).

### 3.2. Meat Fatty Acid Profile

The fatty acid (FA) composition of meat (breast) is summarized in Table 5. In particular, cis-oleic acid (C18:1c) and palmitic acid (C16:0), were the primary FAs present in meat, followed by cis-linoleic acid (C18:2c) and stearic acid (C18:0). Both PS and S influenced all FAs, although season had no effect on C16:1. Regarding PSs, the concentration of C14:0, C18:3n6, C18:3n3, C20:1, C20:2n6 were significantly higher in the ISs. In contrast, significantly elevated contents of C12:0, C15:0, C20:3n6 and EPA were found in meat from the ESs. Meat from DPSs exhibited significantly higher levels of C18:0, C20:4n6, DHA, C24:0 and C24:1. Seasonal differences were also observed, as significantly higher levels were found for C16:0 (spring and summer), C18:0 (winter and summer), C18:1-cis, C18:3n3 (winter and spring), C18:2-cis (autumn and spring), C18:3n6, C24:1 (summer), DHA (autumn and summer). According to the interaction effect (Table S2), in autumn and winter the IS showed significantly higher means for C14:0, C16:1, C18:2-cis, and C18:3n3. In spring the differences between IS and DPS were not statistically significant. The contents of C20:2, C20:3, C20:4 and C24:1 were significantly higher in ES and DPS within every season.

The FA classes (Table 6) provided more details on the effects of PS, S and their interaction. The effect of PS was significant, with meat from DPSs displaying a higher mean PUFA content and lower MUFA content (34.8 ± 0.40% and 27.7 ± 0.50%, respectively). SFA content was significantly lower in ISs (34.1 ± 0.25%), while UFA content was significantly raised (65.9 ± 0.25%). The percentages of ω-3 and ω-6 were significantly elevated in DPS where a reduced ω-6/ω-3 ratio was also recorded (12.0 ± 1.00). Lower means of AI and TI were detected in IS. S had an evident effect on FA classes as higher means were obtained for PUFA (winter and summer), SFA (summer), PUFA/SFA (autumn and spring). At the same time, lower levels were found for UFA (summer), the ω-6/ω-3 ratio (winter), and the indices of atherogenicity (AI) and thrombogenicity (TI) (autumn). The S × PS effect revealed statistically significant differences between PS within each S (Table S3). Breast meat from ES displayed higher MUFA mean (winter, summer), while breast meat from DPS showed elevated means of PUFA (autumn, spring). DPS and ES showed significantly higher SFA means in winter and summer, respectively. DPS also showed higher means ω-3 (spring, summer) and ω-6 (autumn, spring).

The FA composition of meat (thigh) is summarized in Table S4. The PS influenced the FA composition. Meat from ISs showed significantly higher levels of C14:0, C18:3n3, and C20:2n6 while meat from ESs displayed higher levels of C16:0 and C18:1c. DPS meat displayed higher means of C18:0, C20:4n6, DHA, and C24:1. S also affected most of the FAs, as there were significantly elevated levels for C12:0 (winter), C16:0, C18:2t (spring and summer), C18:2c (autumn and spring), and C20:3n6 (autumn). Moreover, the S × PS analysis (see Table S5) showed that IS thigh meat contained higher amounts of C14:0 (winter, spring), C16:1 (spring), C18:1-trans (winter), and C20:2 (autumn, winter, summer), whereas DPS contained higher contents of C18:0 (autumn, winter, spring), C18:2-cis (spring), C20:2 (spring), DHA (all seasons) and C24:1 (autumn, spring, summer). In meat from ESs, greater contents of C18:1-cis (winter, spring, summer), C20:3 (autumn) were recorded.

Additionally, the classes of FA were influenced by PS and S (Table S5). Regarding the PS effect, MUFA content was significantly higher for ES, whereas PUFAs, ω-3 and ω-6 contents were greater for the DPS. The smaller means of AI and TI were detected in DPS and IS, respectively. The ratio of ω-6/ω-3 was increased for the ISs. There were also increased contents of PUFAs and ω-3 (autumn), MUFAs (summer) and PUFA/SFA (autumn), while decreased concentrations were recorded for ω-6 (summer), UFA (winter) and TI (autumn, winter). Several significant differences were observed though the S × PS analysis as well (Table S5). The ISs displayed higher contents of ω-3 (winter) and ω-6/ω-3 (autumn) while the ESs displayed higher contents of MUFA (winter, summer). Lastly, the DPS showed greater means of PUFA (autumn, spring), ω-3 (autumn, spring, summer) and ω-6 (autumn, spring).

### 3.3. Oxidation Stability

The TBARS assay results, presented in Table 7, showed that PS and season effects were significant, with the mean MDA content (mg/Kg) significantly higher in DPSs (0.75 ± 0.02) than in ESs (0.64 ± 0.02) and ISs (0.58 ± 0.02). In addition, MDA content was significantly elevated in winter (0.76 ± 0.02). There was also a significant interaction effect (Table S1), confirming elevated MDA accumulation in meat from ESs and DPSs in autumn, winter and spring.

## 4. Discussion

This study involved six poultry farms to collect and analyze quality parameters of chicken meat. Sampling was performed on a seasonal basis to detect differences between seasons and management practices. The three PSs differed simultaneously across multiple interlinked factors, including genotype, slaughter age and weight, and diet composition/forage availability. Thus, the observed differences in meat quality parameters should be interpreted as outcomes of these integrated systems rather than isolated effects of individual components. According to the results, the influence of PS and S on carcass quality traits was notable. Although most studies have shown higher CW in systems rearing fast-growing broilers, the current study found higher CW in the DPSs. This finding is probably related to the age at which chickens were moved to the slaughterhouse; specifically, those in DPSs had an extended lifespan, which linked to greater growth and, subsequently, greater weight gain. It has previously been shown that low-input systems (free-range) can produce heavier carcasses [19]. Carcasses from ESs exhibited the lowest mean, probably due to reduced total feed intake and the inconsistent availability of forage throughout the production cycle.

It was also noted that CW was affected by the season. It is suggested that flock activity may be reduced in colder seasons, leading to bigger CW, whereas in hotter seasons reduced feed intake may contribute to smaller CW. High temperatures in the shed and the accumulation of anti-nutritional agents, such as mycotoxins in the feed, have been reported as environmental stressors [20]. Additionally, CW varied among PSs within each S, underscoring fluctuations in forage availability throughout the year (DPSs, ESs).

The evaluation of ultimate pH on PM at 24 h post-slaughter aimed to reveal the metabolic condition prior to slaughter. This refers to the handling and transfer of chickens to the slaughterhouse. An increased metabolic rate before slaughter is associated with a post-slaughter decline in carcass pH and is indicative of extensive stress [21]. The ultimate pH is determined by the amount of glycogen stored in muscle and is inversely correlated, as glycogen is ultimately converted to lactic acid, which lowers pH [22]. There were statistically significant differences between PS and S, with ESs displaying a lower mean, suggesting that the transition from outdoors to the slaughterhouse might become more stressful in these systems. A more rapid pH reduction can lead to greater muscle protein denaturation and degradation of the technological properties of meat [23,24]. Da Silva et al. (2017) also found lower mean pH in free-range broiler meat [25]. Winter and spring samplings displayed lower pH values, suggesting that colder temperatures affect the metabolic state of muscle tissue and subsequently the final pH. The interplay of S × PS further showed that the ESs displayed lower means, followed by DPSs within seasons. However most means fell within the normal range of pH at 24 h post-mortem, as previous studies have reported [22]. Similarly, the analysis of ultimate pH on TA revealed a higher mean for the ISs. Winter and spring samplings also showed lower pH means, and a similar interaction effect was observed. The mean pH values between PM and TA were not compared, as each muscle consists of different fiber types (more glycolytic in PM vs. mostly oxidative in TA), with different metabolic pathways that result in different final pH [23].

One of the most crucial observable traits that determines the purchase decision of poultry meat in markets is its color [26]. More specifically, consumers seek chicken filets with a uniform pinkish color, associating it with freshness [27]. Although the differences between ISs, ESs and DPSs for a* and L* values were minor, it is evident that breast meat produced in IS and ES had slightly more redness and appeared to be darker compared to DPSs. Typically, higher redness and lower lightness values correlate with the more developed muscle tissue of older birds [28]; however, this was not observed for the DPSs of the study. It should be noted that the final pH is a trait related to meat color, as breast meat with higher final pH presented higher a* and lower L* values (as in the case of ISs in the current study) [26]. The difference in b* values between PSs might be explained by increased carotenoid consumption through foraging, as observed in the ESs of the current study. This is also supported in previous studies [25]. The same trend appeared for Ch and Hue values. Moreover, the availability of pigments in grass might explain the seasonal fluctuations of these traits (higher in autumn and summer). The interaction effect indicated that PS influenced each S group (a strong effect of the factor PS, responsible for the interaction). The colorimetric traits not only aid the color comparison of carcasses but also explain some technological properties of meat. More specifically, a rise in L* value often results from enhanced light scattering caused by protein degradation within the muscle tissue, leading to the subsequent release of water molecules [14]. This often leads to tender meat; however, in the current study, carcasses from DPS presented both elevated L value and moisture content, but also higher F means. The tougher meat in this case is mainly explained by the increased activity and advanced age [3] of the birds in these PSs. The collagen content also showed significant differences between PSs, further supporting the age-dependent changes in the structure of muscle, in particular the reduced dissolution of collagen in meat from older birds [29]. Carcasses from ES and DPS displayed increased meat firmness, especially in autumn, spring and summer, due to increased mobility in outdoor spaces. It should be noted that the firmness analysis was performed on uncooked meat, limiting the comparability with other studies that performed tenderness evaluation on cooked poultry meat.

The highest fat content (%) in meat was observed in ISs, where fast-growing chicken strains were used. This finding may be explained by the more rapid growth of the adipose tissue in these chickens, in agreement with previous studies [30,31]. Higher means of fat were found in ISs for all seasons. Conversely, the protein content of meat was found to be higher in carcasses from DPSs followed by those from ESs, which is in agreement with previous research involving slow-growing broiler lines [19,32,33]. This trend appeared for all seasons. Regarding the seasonal effect on fat content, the results comply with those from previous studies [34], which reported a higher fat content in hotter seasons. However, the pattern of variability for protein content deviates, as there was no significant reduction in protein (%) in breast meat during hotter seasons.

In poultry, lipid metabolism relies on the exogenous intake of fats and some non-fat sources [30]. This reflects the fatty acid composition of meat. The variations in FAs in breast and thigh meat between PSs suggest that a more balanced diet with controlled feed composition (IS) may be responsible for more favorable FA composition and health indices. Furthermore, the inclusion of minerals and soybean byproducts in the feed may contribute to a more desirable FA profile [35,36]. Several studies have reported similar differences in FA composition and FA groups [37]. The addition of maize, fish oil and rapeseed in the diet of IS birds is associated with an enhanced content of C18:2c, C18:3n3, and C20:1 in breast meat [38,39,40]. The seasonal variation in FAs depends on their availability—or of their precursors—on the grasses that the growing chickens consume while grazing. This reflects the reduced mean contents of C18:2c, C18:3n3 and UFA during summer, in agreement with previous studies [41]. The interaction pattern further describes the greater variation in FAs for the ES and DPS compared to the IS where controlled feeding and uniform feed composition are part of the management practices. Moreover, the fluctuations of PUFA, ω-3 and ω-6 within S may be related to their availability in the forage; for ES and DPS, elevated amounts were detected during autumn, spring and summer. Also it should be mentioned that in such systems the use of these FAs for immunological and metabolic purposes may reduce their deposition in meat [42]. Although several other nutritional indices analyzed (AI, TI, PUFA/SFA) were slightly more favorable in meat from ISs, overall the mean values obtained from all PSs fell within the desired threshold for high-quality meat, as other researchers reported [43].

Shelf life of meat is also an attribute of its quality. Biochemical changes, especially oxidation reactions, occur rapidly in meat, within 3 days after its purchase and during storage conditions [44]. The results of oxidation stability revealed greater MDA content in meat from DPSs and ESs and therefore a reduced shelf life. Meat from ISs displayed lower MDA content. The prolonged age at slaughter in those systems, which is associated with greater growth and muscle development in chickens, might explain the differences between PSs. More specifically, the amount of myoglobin present in the muscle tissue and its containment of iron serves as prooxidant to oxidation reactions. Additionally, the rancidity of meat depends on its PUFAs content, as the double bonds present in PUFAs interact with electrons from adjacent molecules and form oxidation products; therefore, meat richer in PUFAs tends to be more sensitive to oxidation [45]. This is supported in the case of DPSs, where the highest PUFAs mean was also observed. Moreover, environmental stressors may influence the oxidation stability of meat. Cold temperatures may affect chickens before slaughter. The variation in MDA accumulation was more notable in DPSs and ESs than in ISs within seasons, as shown by the S × PS effect.

## 5. Conclusions

This study confirmed the effect of production system and season on broiler meat quality. Production methods and environmental conditions affect the quality of chicken meat. The observed differences among the production systems should be interpreted as outcomes of integrated approaches that encompass interconnected variables. However, the results suggest that no single PS dominated across all evaluated traits, revealing trade-offs in yield and composition. Interestingly, these objective differences do not fully align with common consumer perceptions. Personal beliefs and production narratives may contribute to this perceptual mismatch. Communication strategies bridge this gap by transparently presenting science-based attributes, enabling informed choices.

## Figures and Tables

**Table 1:** Summary of production system main characteristics.

	Production System		
	IS	ES	DPS
Genotype	Ross 308	Hubbard	Sasso silver
Feeding	Concentrates + Supplements	Forages + Supplements	Forages + Supplements
Stocking density (Kg/m^2^)	39	19–22	12
Age at slaughter (days)	38–42	56–70	100
Outdoor access	no	yes	yes

IS = Intensive system; ES = Extensive system; DPS = Dual-purpose system.

**Table 2:** Intensive broiler feed composition.

	Duration (Days) ^1^					
Nutrient component	0–4	5–10	11–15	16–20	21–29	30–42
Crude Protein (%)	21	20.6	20	20.8	22.1	23.9
Crude Fat (%)	6.2	5.7	5.5	5.9	8.5	10.1
Crude Fiber (%)	3.2	3.3	3.7	3.3	3.7	3.5
Crude Ash (%)	5.6	5.7	5.5	5.8	6.2	6.2
Calcium (g)	7.0	7.4	6.6	7.3	7.8	8.3
Phosphorus (g)	5.7	4.8	4.8	4.8	4.3	4.0
Lysine (g)	13.6	13.1	12.9	13.6	14.8	16.7
Methionine (g)	5.8	5.8	5.6	5.7	6.1	6.9
Methionine + Cysteine (g)	9.2	9.2	9.2	9.2	9.7	9.9
Vitamin A (IU)	13,700	13,700	11,300	12,650	14,450	16,850
Vitamin D3 (IU)	2600	2600	3400	3800	4350	4200
Vitamin E (mg)	50	50	50	50	50	50
Vitamin D (µg)	65	65	30	35	65	65
Iron (mg)	51	51	51	57	65	67
Iodine (mg)	2	2	2	2	2	1
Copper (mg)	15	15	16	19	21	25
Manganese (mg)	81	81	79	89	101	118
Zinc (mg)	81	81	72	72	75	72

^1^ Feed formulation according to days; 0–4: prestart; 5–10: starter; 11–15 grower 1; 16–20 grower 2; 21–29: grower 3; 30–42: finisher.

**Table 3:** Dual-purpose feed supplement composition.

Nutrient Component	Value
Moisture %	12.50
Crude protein %	16.50
Crude fat %	5.90
Crude fiber %	5.00
Crude ash %	12.30
Lysine %	0.67
Methionine %	0.27
Calcium %	3.65
Phosphorus %	0.55
Sodium %	0.16
Vitamin A (IU)	10,000
Vitamin D3 (IU)	3000
Vitamin E (IU)	50.00
Iron (mg)	50.00
Iodine (mg)	1.50
Copper (mg)	10.00
Manganese (mg)	80.00
Zinc (mg)	80.00
Selenium (mg)	0.30

**Table 4:** Physicochemical properties and gross chemical composition of chicken breast meat across production systems and seasons.

	PS			S				Significance (*p*-Value)		
Trait	IS	ES	DPS	A	W	Sp	Su	PS	S	S × PS
CW (g)	1750 ± 32.0 ^a^	1649 ± 32.0 ^b^	1828 ± 35.6 ^a^	1870 ± 36.0 ^a^	1827 ± 35.4 ^a^	1575 ± 42.5 ^b^	1697 ± 36.0 ^c^	0.045	0.040	0.033
pH (breast)	5.81 ± 0.02 ^a^	5.66 ± 0.02 ^b^	5.73 ± 0.02 ^c^	5.82 ± 0.02 ^a^	5.67 ± 0.02 ^b^	5.67 ± 0.02 ^b^	5.77 ± 0.02 ^a^	0.011	0.022	0.003
pH (thigh)	6.20 ± 0.02 ^a^	6.07 ± 0.02 ^b^	6.03 ± 0.02 ^b^	6.17 ± 0.02 ^a^	6.02 ± 0.02 ^b^	6.08 ± 0.02 ^b^	6.14 ± 0.02 ^a^	0.014	0.034	0.045
L*	62.8 ± 0.33 ^a^	61.6 ± 0.33 ^b^	64.8 ± 0.34 ^c^	62.5 ± 0.40 ^a^	62.1 ± 0.40 ^a^	63.1 ± 0.40 ^a^	64.6 ± 0.40 ^b^	0.004	0.021	0.032
a*	12.4 ± 0.20 ^a^	12.1 ± 0.20 ^a^	11.0 ± 0.20 ^b^	11.9 ± 0.20 ^a^	11.8 ± 0.20 ^a^	11.7 ± 0.20 ^a^	12.0 ± 0.20 ^a^	0.042	0.597	0.039
b*	7.2 ± 0.20 ^a^	17.6 ± 0.20 ^b^	7.8 ± 0.20 ^a^	11.6 ± 0.30 ^a^	10.6 ± 0.30 ^bc^	10.2 ± 0.30 ^b^	11.1 ± 0.30 ^ac^	0.003	0.031	0.042
Ch	14.4 ± 0.20 ^a^	21.8 ± 0.20 ^b^	13.6 ± 0.20 ^c^	17.2 ± 0.20 ^a^	16.5 ± 0.20 ^b^	15.8 ± 0.20 ^c^	16.8 ± 0.20 ^ab^	0.036	0.044	0.038
Hue	13.4 ± 0.70 ^a^	55.9 ± 0.70 ^b^	35.4 ± 0.80 ^c^	41.9 ± 0.80 ^a^	38.8 ± 0.80 ^b^	40.1 ± 0.90 ^ab^	40.5 ± 0.80 ^ab^	0.010	0.042	0.027
F (N)	82.4 ± 2.30 ^a^	93.3 ± 2.30 ^b^	102.6 ± 2.60 ^c^	95.3 ± 2.70 ^a^	92.6 ± 2.70 ^a^	87.9 ± 3.00 ^a^	95.3 ± 2.70 ^a^	0.037	0.255	0.046
Fat (%)	1.42 ± 0.03 ^a^	0.87 ± 0.03 ^b^	0.25 ± 0.05 ^c^	0.77 ± 0.04 ^a^	0.89 ± 0.04 ^ab^	0.82 ± 0.05 ^ab^	0.91 ± 0.04 ^b^	0.023	0.036	0.040
Protein (%)	23.0 + 0.05 ^a^	23.1 + 0.05 ^a^	23.4 ± 0.05 ^b^	23.2 ± 0.06 ^abc^	23.3 ± 0.06 ^b^	23.0 ± 0.06 ^c^	23.2 ± 0.06 ^b^	0.032	0.040	0.037
Moisture (%)	75.3 ± 0.05 ^a^	75.0 ± 0.05 ^b^	75.9 ± 0.06 ^c^	75.4 ± 0.10 ^a^	75.4 ± 0.10 ^a^	75.6 ± 0.10 ^a^	75.4 ± 0.10 ^a^	0.039	0.216	0.043
Collagen (%)	0.51 ± 0.01 ^a^	0.30 ± 0.01 ^b^	0.08 ± 0.02 ^c^	0.27 ± 0.01 ^a^	0.31 ± 0.02 ^a^	0.29 ± 0.02 ^a^	0.31 ± 0.02 ^a^	0.021	0.185	0.042

Mean ± standard error for quality characteristics of the examined carcasses. Different superscripts (a–c) indicate statistically significant differences at α = 0.05 (*p* ≤ 0.05) among groups of PS or S, according to the LSD criterion. PS = Production system; S = Season; IS = Intensive system; ES = Extensive system; DPS = Dual-Purpose system; A = Autumn; W = Winter; Sp = Spring; Su = Summer; CW = Carcass Weight; L* = Lightness; a* = redness; b* = yellowness; Ch = Chroma; Hue = Hue angle; F = Firmness; S × PS = interaction effect.

**Table 5:** Fatty acid composition (%) of broiler breast meat across production systems and seasons.

	PS			S				Significance (*p*-Value)		
FA	IS	ES	DPS	A	W	Sp	Su	PS	S	S × PS
C12:0	0.87 ± 0.10 ^a^	1.73 ± 0.10 ^b^	1.60 ± 0.10 ^b^	1.51 ± 0.10 ^a^	1.78 ± 0.10 ^b^	1.26 ± 0.10 ^c^	1.04 ± 0.10 ^c^	0.032	0.039	0.046
C14:0	1.48 ± 0.10 ^a^	1.00 ± 0.10 ^b^	1.06 ± 0.10 ^b^	0.80 ± 0.10 ^a^	1.29 ± 0.10 ^b^	1.37 ± 0.10 ^b^	1.25 ± 0.10 ^b^	0.045	0.025	0.007
C15:0	0.46 ± 0.30 ^a^	0.81 ± 0.30 ^b^	0.76 ± 0.35 ^b^	0.76 ± 0.40 ^a^	0.88 ± 0.40 ^b^	0.55 ± 0.40 ^c^	0.51 ± 0.40 ^c^	0.003	0.028	0.030
C16:0	20.4 ± 0.20 ^a^	21.3 ± 0.20 ^b^	20.4 ± 0.20 ^a^	19.8 ± 0.20 ^a^	20.1 ± 0.20 ^a^	21.3 ± 0.20 ^b^	21.4 ± 0.20 ^b^	0.040	0.042	0.047
C16:1	2.90 ± 0.10 ^a^	2.06 ± 0.10 ^b^	1.23 ± 0.10 ^c^	2.03 ± 0.10 ^a^	2.09 ± 0.10 ^a^	2.06 ± 0.10 ^a^	2.03 ± 0.10 ^a^	0.002	0.973	0.041
C18:0	10.0 ± 0.20 ^a^	10.8 ± 0.20 ^b^	11.9 ± 0.20 ^c^	11.0 ± 0.20 ^ab^	10.6 ± 0.20 ^a^	10.2 ± 0.20 ^c^	11.3 ± 0.20 ^b^	0.008	0.045	0.032
C18:1 t	0.29 ± 0.01 ^a^	0.30 ± 0.01 ^a^	0.22 ± 0.01 ^b^	0.28 ± 0.01 ^a^	0.27 ± 0.01 ^a^	0.20 ± 0.02 ^b^	0.34 ± 0.01 ^c^	0.041	0.044	0.046
C18:1 c	29.9 ± 0.40 ^a^	30.3 ± 0.40 ^a^	24.3 ± 0.50 ^b^	27.5 ± 0.50 ^a^	28.9 ± 0.50 ^b^	28.8 ± 0.60 ^ab^	27.5 ± 0.50 ^a^	0.013	0.036	0.020
C18:2 t	0.18 ± 0.01 ^ab^	0.21 ± 0.01 ^a^	0.15 ± 0.02 ^b^	0.17 ± 0.01 ^a^	0.15 ± 0.01 ^a^	0.10 ± 0.02 ^b^	0.29 ± 0.02 ^c^	0.045	0.039	0.045
C18:2 c	22.5 ± 0.30 ^a^	17.5 ± 0.30 ^b^	20.0 ± 0.30 ^c^	20.3 ± 0.30 ^ab^	19.5 ± 0.30 ^ac^	21.2 ± 0.40 ^b^	18.7 ± 0.30 ^c^	0.031	0.038	0.042
C18:3 n6	0.23 ± 0.01 ^a^	0.21 ± 0.02 ^ab^	0.17 ± 0.02 ^b^	0.21 ± 0.10 ^a^	0.20 ± 0.20 ^a^	0.13 ± 0.20 ^b^	0.28 ± 0.20 ^c^	0.027	0.032	0.046
C18:3 n3	1.15 ± 0.04 ^a^	0.50 ± 0.04 ^b^	0.80 ± 0.05 ^c^	0.80 ± 0.05 ^a^	0.97 ± 0.05 ^b^	0.82 ± 0.06 ^ab^	0.65 ± 0.05 ^c^	0.031	0.043	0.040
C20:1	0.56 ± 0.02 ^a^	0.45 ± 0.02 ^b^	0.30 ± 0.02 ^c^	0.39 ± 0.02 ^a^	0.41 ± 0.02 ^a^	0.27 ± 0.02 ^b^	0.68 ± 0.02 ^c^	0.040	0.025	0.037
C:20:2 n6	0.89 ± 0.03 ^a^	0.71 ± 0.03 ^b^	0.55 ± 0.04 ^a^	0.73 ± 0.03 ^ab^	0.63 ± 0.03 ^a^	0.70 ± 0.04 ^a^	0.83 ± 0.03 ^b^	0.003	0.030	0.022
C:20:3 n6	0.99 ± 0.04 ^a^	1.26 ± 0.04 ^b^	1.05 ± 0.04 ^a^	1.20 ± 0.04 ^ab^	1.08 ± 0.04 ^a^	0.88 ± 0.05 ^c^	1.26 ± 0.04 ^b^	0.032	0.045	0.042
C20:4 n6	4.70 ± 0.20 ^a^	6.84 ± 0.20 ^b^	10.43 ± 0.30 ^c^	7.98 ± 0.30 ^a^	7.02 ± 0.30 ^b^	6.81 ± 0.30 ^b^	7.45 ± 0.30 ^ab^	0.002	0.027	0.023
EPA	0.40 ± 0.02 ^a^	0.51 ± 0.02 ^b^	0.33 ± 0.02 ^c^	0.37 ± 0.03 ^ab^	0.42 ± 0.02 ^a^	0.33 ± 0.03 ^b^	0.53 ± 0.03 ^c^	0.026	0.041	0.048
DHA	0.50 ± 0.05 ^a^	0.97 ± 0.05 ^b^	1.74 ± 0.05 ^c^	1.22 ± 0.05 ^a^	1.01 ± 0.05 ^bc^	0.89 ± 0.06 ^b^	1.15 ± 0.05 ^ac^	0.015	0.030	0.028
C24:0	1.40 ± 0.10 ^a^	1.70 ± 0.10 ^b^	1.78 ± 0.10 ^b^	1.81 ± 0.10 ^a^	1.66 ± 0.10 ^a^	1.30 ± 0.10 ^b^	1.73 ± 0.10 ^a^	0.033	0.041	0.039
C24:1	0.86 ± 0.04 ^a^	1.07 ± 0.04 ^b^	1.70 ± 0.05 ^c^	1.27 ± 0.05 ^a^	1.12 ± 0.05 ^b^	1.00 ± 0.06 ^b^	1.40 ± 0.05 ^c^	0.004	0.036	0.026

Fatty acids (%) in chicken breast (mean ± standard error). Different superscripts (a–c) indicate statistically significant differences at α = 0.05 (*p* ≤ 0.05) among groups of PS or S, according to the LSD criterion. FA = Fatty acid; PS = Production system; S = Season; IS = Intensive system; ES = Extensive system; DPS = Dual-Purpose system; A = Autumn; W = Winter; Sp = Spring; Su = Summer; S × PS = interaction effect; C12:0 = lauric; C14:0 = myristic; C15:0 = pentadecanoic; C16:0 = palmitic; C16:1 = palmitoleic; C18:0 = stearic; C18:1t = trans-oleic; C18:1c = cis-oleic; C18:2t = trans-linoleic; C18:2c = cis-linoleic; C18:3n6 = gama linolenic; C18:3n3 = alpha linolenic; C20:1 = cis-gondoic; C20:2n6 = cis-eicosadienoic; C20:3n6 = dihomo-gama linolenic; C20:4n6 = arachidonic; EPA = eicosapentaenoic acid; DHA = docosahexaenoic acid; C24:0 = lignoceric; C24:1 = nervonic acid.

**Table 6:** Effect of production system and season on fatty-acid classes of chicken breast meat (% of total fatty acids).

	PS			S				Significance (*p*-Value)		
FA Class	IS	ES	DPS	A	W	Sp	Su	PS	S	S × PS
MUFA	34.5 ± 0.40 ^a^	34.2 ± 0.40 ^a^	27.7 ± 0.50 ^b^	31.5 ± 0.50 ^a^	32.8 ± 0.50 ^a^	32.4 ± 0.60 ^a^	31.9 ± 0.50 ^a^	0.042	0.280	0.046
PUFA	31.4 ± 0.40 ^a^	28.5 ± 0.40 ^b^	34.8 ± 0.40 ^c^	32.9 ± 0.40 ^a^	30.9 ± 0.40 ^b^	31.7 ± 0.50 ^ab^	30.9 ± 0.40 ^b^	0.033	0.044	0.036
SFA	34.1 ± 0.25 ^a^	37.3 ± 0.25 ^b^	37.5 ± 0.30 ^b^	35.6 ± 0.30 ^a^	36.3 ± 0.30 ^a^	35.9 ± 0.35 ^a^	37.3 ± 0.30 ^b^	0.004	0.037	0.022
UFA	65.9 ± 0.25 ^a^	62.7 ± 0.25 ^b^	62.5 ± 0.30 ^b^	64.4 ± 0.30 ^a^	63.7 ± 0.30 ^a^	64.1 ± 0.30 ^a^	62.7 ± 0.30 ^b^	0.006	0.036	0.030
PUFA/SFA	0.93 ± 0.01 ^a^	0.77 ± 0.01 ^b^	0.93 ± 0.01 ^a^	0.93 ± 0.01 ^a^	0.86 ± 0.01 ^bc^	0.89 ± 0.01 ^ab^	0.83 ± 0.01 ^c^	0.023	0.034	0.028
ω-3	1.98 ± 0.10 ^a^	1.96 ± 0.10 ^a^	2.85 ± 0.10 ^b^	2.36 ± 0.10 ^a^	2.40 ± 0.10 ^a^	2.03 ± 0.10 ^b^	2.26 ± 0.10 ^ab^	0.034	0.042	0.039
ω-6	29.4 ± 0.30 ^a^	26.6 ± 0.30 ^b^	32.0 ± 0.40 ^c^	30.5 ± 0.40 ^a^	28.5 ± 0.40 ^b^	29.7 ± 0.45 ^ac^	28.6 ± 0.40 ^bc^	0.037	0.046	0.042
ω-6/ω-3	18.5 ± 1.00 ^a^	14.2 ± 1.00 ^b^	12.9 ± 1.00 ^b^	15.4 ± 1.00 ^a^	12.2 ± 1.00 ^b^	16.2 ± 1.00 ^a^	16.9 ± 1.00 ^a^	0.039	0.041	0.043
AI	0.41 ± 0.01 ^a^	0.43 ± 0.01 ^b^	0.42 ± 0.01 ^ab^	0.38 ± 0.01 ^a^	0.43 ± 0.01 ^b^	0.44 ± 0.01 ^b^	0.44 ± 0.01 ^b^	0.044	0.038	0.042
TI	0.84 ± 0.01 ^a^	0.91 ± 0.01 ^b^	0.87 ± 0.01 ^c^	0.83 ± 0.01 ^a^	0.85 ± 0.01 ^a^	0.89 ± 0.01 ^b^	0.93 ± 0.01 ^c^	0.025	0.031	0.045

FA class (%) in chicken breast (mean ± standard error). Different superscripts (a–c) indicate statistically significant differences at α = 0.05 (*p* ≤ 0.05) among groups of PS or S, according to the LSD criterion. FA = Fatty acid; PS = Production system; S = Season; IS = Intensive system; ES = Extensive system; DPS = Dual-Purpose system; A = Autumn; W = Winter; Sp = Spring; Su = Summer; S × PS = interaction effect. MUFA = monounsaturated fatty acid; PUFA = polyunsaturated fatty acids; SFA = total saturated fatty acids; UFA = total unsaturated fatty acids; ω-3 = omega 3 fatty acids; ω-6 = omega 6 fatty acids; AI = Atherogenicity index; TI = Thrombogenicity index. AI and TI were calculated according to Ulbricht and Southgate (1991) [18]

**Table 7:** Oxidative stability of chicken breast meat across production systems and seasons.

	PS			S				Significance (*p*-Value)		
Trait	IS	ES	DPS	A	W	Sp	Su	PS	S	S × PS
MDA (mg/Kg)	0.58 ± 0.02 ^a^	0.64 ± 0.02 ^b^	0.75 ± 0.02 ^c^	0.65 ± 0.02 ^a^	0.76 ± 0.02 ^b^	0.60 ± 0.02 ^a^	0.62 ± 0.02 ^a^	0.002	0.035	0.028

Mean ± standard error for MDA content. Different superscripts (a–c) indicate statistically significant differences at α = 0.05 (*p* ≤ 0.05) among groups of PS or S, according to the LSD criterion. PS = Production system; S = Season; IS = Intensive system; ES = Extensive system; DPS = Dual-Purpose system; A = Autumn; W = Winter; Sp = Spring; Su = Summer; S × PS = interaction effect.

## Data Availability

The original contributions presented in this study are included in the article, and further inquiries can be directed to the corresponding author.

## Supplementary Materials

The following supporting information can be downloaded at: https://www.mdpi.com/article/10.3390/ani16020272/s1, Table S1: Interaction effect of PS and S on chicken meat physicochemical properties; Table S2: Interaction effect of PS and S on breast FA profile; Table S3: Interaction effect of PS and S on breast FA classes and nutritional indices; Table S4: Fatty acid composition (%) on broiler thigh; Table S5: Effect of PS and S on FA classes of chicken thigh; Table S6: Interaction effect of PS and S on thigh FA profile; Table S7: Interaction effect of PS and S on thigh FA classes and nutritional indices.

## Abbreviations

The following abbreviations are used in this manuscript:

PS	Production system
IS	Intensive system
ES	Extensive system
DPS	Dual-purpose system
PM	Pectoralis major
TA	Tibialis anterior
L*	Lightness
a*	Redness
b*	Yellowness
Ch	Chroma
Hue	Hue angle
MUFA	Monounsaturated fatty acid
PUFA	Polyunsaturated fatty acid
UFA	Unsaturated fatty acid
SFA	Saturated fatty acid
AI	Atherogenicity index
TI	Thrombogenicity index
MDA	Malondialdehyde
