# Supplementary material for: Assessment of Fast-Growing and Dual-Purpose Chicken Meat Quality Characteristics in Different Production Systems
Source: Animals (Basel). 2026 Jan 16;16(2):272. doi: 10.3390/ani16020272 (PMC12837140; doi:10.3390/ani16020272)
Supplement: Supplementary file 1 [file animals-16-00272-s001.zip › animals-4069547-supplementary.pdf]

Assessment of fast-growing and dual-purpose chicken meat quality characteristics in different production systems

Supplementary

Table S1. Interaction effect of PS and S on chicken meat physicochemical properties

	A			W			Sp			Su		
Trait	IS	ES	DPS	IS	ES	DPS	IS	ES	DPS	IS	ES	DPS
CW (g)	1661 ± 64.6^{a}	2044 ± 61.2^{b}	1903 ± 61.2^{b}	1686 ± 61.2^{a}	1617 ± 61.2^{a}	2179 ± 61.2^{b}	1712 ± 61.2^{a}	1447 ± 61.2^{b}	1566 ± 93.5^{ab}	1941 ± 61.2^{a}	1487 ± 64.6^{b}	1664 ± 61.2^{b}
pHb	5.96 ± 0.04^{a}	5.75 ± 0.03^{b}	5.75 ± 0.03^{b}	5.78 ± 0.03^{a}	5.62 ± 0.03^{b}	5.62 ± 0.03^{b}	5.65 ± 0.03^{a}	5.60 ± 0.03^{a}	5.77 ± 0.05^{b}	5.86 ± 0.03^{a}	5.68 ± 0.03^{b}	5.77 ± 0.03^{ab}
pHt	6.34 ± 0.04^{a}	6.13 ± 0.04^{b}	6.04 ± 0.04^{b}	6.22 ± 0.04^{a}	6.00 ± 0.04^{b}	5.85 ± 0.04^{c}	6.13 ± 0.04^{a}	5.98 ± 0.04^{b}	6.12 ± 0.05^{a}	6.12 ± 0.04^{a}	6.18 ± 0.04^{a}	6.12 ± 0.04^{a}
L	62.7 ± 0.70^{a}	61.5 ± 0.70^{a}	63.3 ± 0.70^{a}	61.6 ± 0.70^{a}	60.5 ± 0.70^{a}	64.2 ± 0.70^{b}	63.4 ± 0.70^{a}	61.9 ± 0.70^{a}	63.9 ± 1.0^{a}	63.6 ± 0.70^{a}	62.5 ± 0.70^{a}	67.6 ± 0.70^{b}
a	12.7 ± 0.30^{a}	12.4 ± 0.30^{a}	10.6 ± 0.30^{b}	12.3 ± 0.30^{a}	11.5 ± 0.30^{a}	11.5 ± 0.30^{a}	12.4 ± 0.30^{a}	11.7 ± 0.30^{ab}	10.9 ± 0.50^{b}	12.3 ± 0.30^{a}	12.9 ± 0.30^{a}	10.8 ± 0.30^{b}
b	6.9 ± 0.50^{a}	18.8 ± 0.50^{b}	9.0 ± 0.50^{c}	6.5 ± 0.50^{a}	18.7 ± 0.50^{b}	6.7 ± 0.50^{a}	7.2 ± 0.50^{a}	14.7 ± 0.50^{b}	8.6 ± 0.70^{a}	7.9 ± 0.50^{a}	18.3 ± 0.50^{b}	7.0 ± 0.50^{a}
Chr	14.6 ± 0.40^{a}	23.1 ± 0.40^{b}	14.1 ± 0.40^{a}	14.0 ± 0.40^{a}	22.1 ± 0.40^{b}	13.4 ± 0.40^{a}	14.4 ± 0.40^{a}	19.0 ± 0.40^{b}	13.9 ± 0.60^{a}	14.7 ± 0.40^{a}	22.8 ± 0.40^{b}	13.0 ± 0.40^{c}
Hue	28.7 ± 1.40^{a}	56.8 ± 1.40^{b}	30.3 ± 1.40^{c}	27.5 ± 1.40^{a}	58.6 ± 1.40^{b}	30.3 ± 1.40^{a}	30.3 ± 1.40^{a}	52.0 ± 1.40^{b}	37.9 ± 2.0^{c}	32.5 ± 1.40^{a}	56.1 ± 1.40^{b}	32.7 ± 1.40^{a}
Firmness	83.1 ± 5.00^{a}	106.3 ± 5.00^{b}	96.6 ± 5.00^{b}	87.9 ± 5.00^{a}	94.4 ± 5.00^{a}	95.5 ± 5.00^{a}	78.8 ± 5.00^{a}	88.0 ± 5.00^{ab}	96.8 ± 7.00^{b}	79.9 ± 5.00^{a}	84.5 ± 5.00^{a}	121.5 ± 5.00^{b}
MDA (mg.Kg)	0.51 ± 0.04^{a}	0.59 ± 0.04^{a}	0.84 ± 0.04^{b}	0.60 ± 0.04^{a}	0.79 ± 0.04^{b}	0.90 ± 0.04^{c}	0.52 ± 0.04^{a}	0.62 ± 0.04^{b}	0.65 ± 0.05^{b}	0.68 ± 0.04^{a}	0.57 ± 0.04^{b}	0.62 ± 0.04^{ab}
Fat (%)	1.41 ± 0.10^{a}	0.76 ± 0.10^{b}	0.15 ± 0.10^{c}	1.43 ± 0.10^{a}	0.95 ± 0.10^{b}	0.28 ± 0.10^{c}	1.42 ± 0.10^{a}	0.80 ± 0.10^{b}	0.24 ± 0.10^{c}	1.42 ± 0.10^{a}	0.99 ± 0.10^{b}	0.31 ± 0.10^{c}
Protein (%)	23.0 ± 0.10^{a}	23.1 ± 0.10^{b}	23.5 ± 0.10^{b}	23.2 ± 0.10^{a}	23.1 ± 0.10^{a}	23.5 ± 0.10^{b}	23.0 ± 0.10^{a}	23.0 ± 0.10^{a}	23.3 ± 0.15^{b}	22.9 ± 0.10^{a}	23.3 ± 0.10^{b}	23.4 ± 0.10^{b}
Moisture (%)	75.3 ± 0.10^{a}	75.0 ± 0.10^{a}	76.0 ± 0.10^{b}	75.3 ± 0.10^{a}	75.1 ± 0.10^{a}	75.6 ± 0.10^{b}	75.3 ± 0.10^{a}	75.2 ± 0.10^{a}	76.1 ± 0.10^{b}	75.4 ± 0.10^{a}	74.8 ± 0.10^{b}	76.0 ± 0.10^{c}
Collagen (%)	0.51 ± 0.02^{a}	0.25 ± 0.02^{b}	0.05 ± 0.02^{c}	0.51 ± 0.02^{a}	0.32 ± 0.03^{b}	0.11 ± 0.03^{c}	0.51 ± 0.02^{a}	0.29 ± 0.03^{b}	0.08 ± 0.04^{c}	0.51 ± 0.02^{a}	0.33 ± 0.03^{b}	0.08 ± 0.03^{c}

Mean ± standard error for external quality characteristics of the examined eggs. Different superscripts (a-c) indicate statistically significant differences at α=0.05 (p≤0.05) among groups of PS within age seasons, according to the LSD criterion. *: $p < 0.05$; PS = Production system; S = Season; IS = Intensive system; ES = Extensive system; DPS = Dual- Purpose system; A= Autumn; W = Winter; Sp = Spring; Su = Summer; CW = Carcass Weight; pHb = pH of breast; pHt = pH of thigh; L = Lightness; a = redness; b = yellowness; Ch = Chroma; Hue = Hue angle; F = Firmness

Table S2. Interaction effect of PS and S on breast FA profile

	A			W			Sp			Su		
FA	IS	ES	DPS	IS	ES	DPS	IS	ES	DPS	IS	ES	DPS
C12:0	0.89 ± 0.14^{a}	1.64 ± 0.14^{b}	1.99 ± 0.13^{b}	1.10 ± 0.13^{a}	2.21 ± 0.13^{b}	2.03 ± 0.13^{b}	0.84 ± 0.13^{a}	1.83 ± 0.13^{b}	1.10 ± 0.20^{a}	0.65 ± 0.13^{a}	1.23 ± 0.14^{b}	1.24 ± 0.13^{b}

C14:0	1.51 ± 0.11^{a}	0.52 ± 0.11^{b}	0.33 ± 0.11^{b}	1.41 ± 0.11^{a}	1.04 ± 0.11^{b}	1.42 ± 0.11^{a}	1.91 ± 0.11^{a}	1.19 ± 0.11^{b}	1.02 ± 0.16^{b}	1.07 ± 0.11^{a}	1.23 ± 0.11^{ab}	1.45 ± 0.11^{b}
C15:0	0.36 ± 0.10^{a}	0.89 ± 0.10^{b}	1.02 ± 0.10^{b}	0.64 ± 0.10^{a}	1.01 ± 0.10^{b}	0.98 ± 0.10^{b}	0.38 ± 0.10^{a}	0.77 ± 0.10^{b}	0.50 ± 0.10^{a}	0.46 ± 0.10^{a}	0.56 ± 0.10^{a}	0.53 ± 0.10^{a}
C16:0	19.3 ± 0.30^{a}	20.4 ± 0.30^{b}	19.7 ± 0.30^{ab}	19.6 ± 0.30^{a}	20.4 ± 0.30^{a}	20.4 ± 0.30^{a}	21.4 ± 0.30^{a}	21.6 ± 0.30^{a}	20.9 ± 0.50^{a}	21.1 ± 0.30^{a}	22.6 ± 0.30^{b}	20.5 ± 0.30^{a}
C16:1	2.55 ± 0.20^{a}	2.20 ± 0.20^{a}	1.40 ± 0.20^{b}	2.89 ± 0.20^{a}	2.05 ± 0.20^{b}	1.33 ± 0.20^{c}	3.13 ± 0.20^{a}	2.00 ± 0.20^{b}	1.10 ± 0.27^{c}	2.94 ± 0.20^{a}	2.03 ± 0.20^{b}	1.12 ± 0.20^{c}
C18:0	9.54 ± 0.30^{a}	10.90 ± 0.30^{b}	12.54 ± 0.30^{c}	9.20 ± 0.30^{a}	10.56 ± 0.30^{b}	11.96 ± 0.30^{c}	8.79 ± 0.30^{a}	10.36 ± 0.30^{b}	11.30 ± 0.50^{b}	10.69 ± 0.30^{a}	11.42 ± 0.30^{ab}	11.92 ± 0.30^{b}
C18:1t	0.25 ± 0.20^{a}	0.33 ± 0.20^{b}	0.25 ± 0.20^{a}	0.35 ± 0.20^{a}	0.25 ± 0.20^{b}	0.22 ± 0.20^{b}	0.19 ± 0.20^{a}	0.25 ± 0.20^{b}	0.15 ± 0.30^{a}	0.38 ± 0.20^{a}	0.37 ± 0.20^{a}	0.27 ± 0.20^{b}
C18:1c	31.0 ± 1.00^{a}	28.4 ± 1.00^{b}	23.1 ± 1.00^{c}	28.9 ± 1.00^{a}	32.6 ± 1.00^{b}	25.5 ± 1.00^{c}	32.1 ± 1.00^{a}	30.6 ± 1.00^{a}	23.6 ± 1.00^{b}	27.7 ± 1.00^{a}	29.8 ± 1.00^{a}	25.1 ± 1.00^{b}
C18:2t	0.14 ± 0.02^{a}	0.22 ± 0.02^{b}	0.16 ± 0.02^{ab}	0.23 ± 0.02^{a}	0.11 ± 0.03^{b}	0.12 ± 0.02^{b}	0.10 ± 0.02^{a}	0.13 ± 0.03^{a}	0.06 ± 0.03^{a}	0.26 ± 0.02^{a}	0.36 ± 0.03^{b}	0.26 ± 0.03^{a}
C18:2c	23.8 ± 0.50^{a}	18.5 ± 0.50^{b}	18.6 ± 0.50b	23.5 ± 0.50^{a}	16.7 ± 0.50^{b}	18.3 ± 0.50^{c}	22.0 ± 0.50^{a}	18.2 ± 0.50^{b}	23.3 ± 0.80^{a}	20.9 ± 0.50^{a}	16.5 ± 0.50^{b}	18.5 ± 0.50^{c}
C18:3n6	0.22 ± 0.02^{a}	0.21 ± 0.02^{a}	0.19 ± 0.03^{a}	0.26 ± 0.02^{a}	0.18 ± 0.03^{b}	0.15 ± 0.03^{b}	0.16 ± 0.02^{a}	0.14 ± 0.03^{ab}	0.10 ± 0.03^{b}	0.27 ± 0.02^{a}	0.32 ± 0.03^{a}	0.25 ± 0.03^{a}
C18:3n3	1.09 ± 0.10^{a}	0.57 ± 0.10^{b}	0.74 ± 0.10^{b}	1.77 ± 0.10^{a}	0.55 ± 0.10^{b}	0.60 ± 0.10^{b}	0.95 ± 0.10^{a}	0.51 ± 0.10^{b}	0.99 ± 0.10^{a}	0.78 ± 0.10^{a}	0.38 ± 0.10^{b}	0.80 ± 0.10^{a}
C20:1	0.42 ± 0.04^{a}	0.48 ± 0.04^{a}	0.26 ± 0.04^{b}	0.49 ± 0.04^{a}	0.47 ± 0.04^{a}	0.27 ± 0.04^{b}	0.41 ± 0.04^{a}	0.34 ± 0.04^{a}	0.10 ± 0.05^{b}	0.91 ± 0.04^{a}	0.51 ± 0.04^{b}	0.63 ± 0.04^{c}
C:20:2n6	0.84 ± 0.10^{a}	0.75 ± 0.10^{a}	0.61 ± 0.10^{b}	0.87 ± 0.10^{a}	0.62 ± 0.10^{b}	0.40 ± 0.10^{c}	0.63 ± 0.10^{a}	0.91 ± 0.10^{b}	0.49 ± 0.10^{a}	1.21 ± 0.10^{a}	0.57 ± 0.10^{b}	0.70 ± 0.10^{b}
C:20:3n6	0.88 ± 0.10^{a}	1.40 ± 0.10^{b}	1.28 ± 0.10^{b}	1.00 ± 0.10^{a}	1.24 ± 0.10^{b}	1.00 ± 0.10^{a}	0.75 ± 0.10^{a}	1.20 ± 0.10^{b}	0.71 ± 0.10^{a}	1.32 ± 0.10^{a}	1.23 ± 0.10^{a}	1.21 ± 0.10^{a}
C20:4n6	4.60 ± 0.50^{a}	7.76 ± 0.50^{b}	11.6 ± 0.50^{c}	4.60 ± 0.50^{a}	6.32 ± 0.50^{b}	10.1 ± 0.50^{c}	3.83 ± 0.50^{a}	6.27 ± 0.50^{b}	10.3 ± 0.70^{c}	5.67 ± 0.50^{a}	7.00 ± 0.50^{b}	9.68 ± 0.50^{c}
EPA	0.24 ± 0.06^{a}	0.50 ± 0.04^{b}	0.37 ± 0.04^{a}	0.44 ± 0.04^{a}	0.52 ± 0.04^{a}	0.31 ± 0.04^{b}	0.39 ± 0.04^{a}	0.45 ± 0.04^{a}	0.13 ± 0.07^{b}	0.53 ± 0.04^{a}	0.55 ± 0.04^{a}	0.51 ± 0.04^{a}
DHA	0.34 ± 0.10^{a}	1.20 ± 0.10^{b}	2.14 ± 0.10^{c}	0.52 ± 0.10^{a}	0.95 ± 0.10^{b}	1.60 ± 0.10^{c}	0.35 ± 0.10^{a}	0.83 ± 0.10^{b}	1.50 ± 0.10^{c}	0.77 ± 0.10^{a}	0.93 ± 0.10^{a}	1.75 ± 0.10^{b}
C24:0	1.29 ± 0.12^{a}	2.02 ± 0.12^{b}	2.1 ± 0.12^{b}	1.37 ± 0.12^{a}	1.63 ± 0.12^{a}	1.97 ± 0.12^{b}	0.98 ± 0.12^{a}	1.48 ± 0.12^{b}	1.37 ± 0.19^{ab}	1.88 ± 0.12^{a}	1.62 ± 0.12^{a}	1.69 ± 0.12^{a}
C24:1	0.62 ± 0.10^{a}	1.30 ± 0.10^{b}	1.89 ± 0.10^{c}	0.96 ± 0.10^{a}	0.98 ± 0.10^{a}	1.42 ± 0.10^{b}	0.65 ± 0.10^{a}	0.95 ± 0.10^{b}	1.40 ± 0.10^{c}	1.20 ± 0.10^{a}	1.07 ± 0.10^{a}	1.94 ± 0.10^{b}

Fatty acids (%) in chicken breast (mean ± standard error). Different superscripts (a-c) indicate statistically significant differences at α=0.05 ($p \leq 0.05$) among groups of PS or S, according to the LSD criterion. *: $p < 0.05$; FA = Fatty acid; PS = Production system; S = Season; IS = Intensive system; ES = Extensive system; DPS = Dual- Purpose system; A= Autumn; W = Winter; Sp = Spring; Su = Summer; C12:0 = lauric; C14:0 = myristic; C15:0 = pentadecanoic; C16:0 = palmitic; C16:1 = palmitoleic; C18:0 = stearic; C18:1t =trans-oleic; C18:1c = cis-oleic; C18:2t = trans- linoleic; C18:2c = cis- linoleic; C18:3n6 = gama linolenic; C18:3n3 = alpha linolenic; C20:1 = cis- gondoic; C20:2n6 = cis-eicosadienoic ; C20:3n6 = dihomo- gama linolenicC20:4n6 = arachidonic; EPA = eicosapentaenoic acid; DHA = docosahexaenoic acid; C24:0 = lignoceric; C24:1 = nervonic acid.

Table S3. Interaction effect of PS and S on breast FA classes and nutritional indices

	**A**			**W**			**Sp**			**Su**		
FA class	IS	ES	DPS	IS	ES	DPS	IS	ES	DPS	IS	ES	DPS
MUFA	35.0 ± 1.00^{a}	32.7 ± 1.00^{a}	26.8 ± 1.00^{b}	33.6 ± 1.00^{a}	36.1 ± 1.00^{b}	28.7 ± 1.00^{c}	36.6 ± 1.00^{a}	34.2 ± 1.00^{b}	26.3 ± 1.00^{c}	32.8 ± 1.00^{a}	33.8 ± 1.00^{a}	29.0 ± 1.00^{b}

PUFA	32.1 ± 1.00^{a}	31.0 ± 1.00^{a}	35.6 ± 1.00^{b}	33.2 ± 1.00^{a}	27.0 ± 1.00^{b}	32.5 ± 1.00^{a}	29.0 ± 1.00^{a}	28.5 ± 1.00^{a}	37.5 ± 1.00^{b}	31.4 ± 1.00^{a}	27.6 ± 1.00^{b}	33.7 ± 1.00^{c}
SFA	32.9 ± 0.50^{a}	36.3 ± 0.50^{b}	37.6 ± 0.50^{b}	33.2 ± 0.50^{a}	36.9 ± 0.50^{b}	38.8 ± 0.50^{c}	34.3 ± 0.50^{a}	37.3 ± 0.50^{b}	36.2 ± 0.70^{b}	35.8 ± 0.50^{a}	38.6 ± 0.50^{b}	37.4 ± 0.50^{b}
UFA	67.1 ± 0.50^{a}	63.7 ± 0.50^{b}	62.4 ± 0.50^{b}	66.8 ± 0.50^{a}	63.1 ± 0.50^{b}	61.2 ± 0.50^{c}	65.7 ± 0.50^{a}	62.7 ± 0.50^{b}	63.8 ± 0.80^{b}	64.2 ± 0.50^{a}	61.4 ± 0.50^{b}	62.6 ± 0.50^{b}
ω-3	1.63 ± 0.15^{a}	2.21 ± 0.15^{b}	3.25 ± 0.15^{c}	2.73 ± 0.15^{a}	2.02 ± 0.15^{b}	2.45 ± 0.15^{a}	1.67 ± 0.15^{a}	1.80 ± 0.15^{a}	2.61 ± 0.20^{b}	1.88 ± 0.15^{a}	1.83 ± 0.15^{a}	3.06 ± 0.15^{b}
ω-6	30.5 ± 1.00^{a}	28.8 ± 1.00^{a}	32.3 ± 1.00^{b}	30.4 ± 1.00^{a}	25.0 ± 1.00^{b}	30.1 ± 1.00^{a}	27.4 ± 1.00^{a}	26.7 ± 1.00^{a}	34.9 ± 1.00^{b}	29.5 ± 1.00^{a}	25.7 ± 1.00^{b}	30.6 ± 1.00^{a}
ω-6.ω-3	22.5 ± 1.20^{a}	13.4 ± 1.20^{b}	10.3 ± 1.20^{b}	11.1 ± 1.20^{a}	12.8 ± 1.20^{a}	12.6 ± 1.20^{a}	17.8 ± 1.20^{a}	15.1 ± 1.20^{a}	15.9 ± 1.90^{a}	22.6 ± 1.20^{a}	15.3 ± 1.20^{b}	12.7 ± 1.20^{b}
PUFA/SFA	0.99 ± 0.03^{a}	0.86 ± 0.03^{b}	0.95 ± 0.02^{a}	1.01 ± 0.02^{a}	0.73 ± 0.02^{b}	0.84 ± 0.02^{c}	0.86 ± 0.02^{a}	0.77 ± 0.02^{b}	1.04 ± 0.04^{c}	0.88 ± 0.02^{a}	0.71 ± 0.02^{b}	0.90 ± 0.02^{a}
AI	0.39 ± 0.01^{a}	0.38 ± 0.01^{a}	0.37 ± 0.01^{a}	0.40 ± 0.01^{a}	0.43 ± 0.01^{a}	0.46 ± 0.01^{b}	0.46 ± 0.01^{a}	0.45 ± 0.01^{ab}	0.41 ± 0.02^{b}	0.41 ± 0.01^{a}	0.47 ± 0.01^{b}	0.44 ± 0.01^{b}
TI	0.81 ± 0.02^{a}	0.85 ± 0.02^{a}	0.83 ± 0.02^{a}	0.75 ± 0.02^{a}	0.87 ± 0.02^{b}	0.92 ± 0.02^{b}	0.88 ± 0.02^{a}	0.93 ± 0.02^{a}	0.87 ± 0.02^{a}	0.91 ± 0.02^{a}	1.00 ± 0.02^{b}	0.87 ± 0.02^{a}

FA class (%) in chicken breast (mean ± standard error). Different superscripts (a-c) indicate statistically significant differences at α=0.05 (p≤0.05) among groups of PS, according to the LSD criterion. *: p < 0.05; FA = Fatty acid; PS = Production system; S = Season; IS = Intensive system; ES = Extensive system; DPS = Dual- Purpose system; A= Autumn; W = Winter; Sp = Spring; Su = Summer; MUFA = monounsaturated fatty acid; PUFA = polyunsaturated fatty acids; SFA = total saturated fatty acids; UFA = total unsaturated fatty acid; ω-3 = omega 3 fatty acids; ω-6 = omega 6 fatty acids; AI = Atherogenicity index; TI = Thrombogenicity index.

Table S4. Fatty acid composition (%) on broiler thigh

	**PS**			**S**				**Significance (*p*-value)**		
FA	IS	ES	DPS	A	W	Sp	Su	PS	S	S x PS
C12:0	0.56 ± 0.04^{a}	0.80 ± 0.04^{b}	0.76 ± 0.05^{b}	0.73 ± 0.05^{a}	1.07 ± 0.05^{b}	0.47 ± 0.06^{c}	0.56 ± 0.05^{c}	*	*	*
C14:0	1.37 ± 0.04^{a}	1.00 ± 0.04^{b}	0.78 ± 0.05^{c}	1.07 ± 0.05^{ab}	1.03 ± 0.05^{a}	1.14 ± 0.06^{a}	0.96 ± 0.05^{b}	*	*	*
C15:0	0.40 ± 0.02^{ab}	0.43 ± 0.02^{a}	0.34 ± 0.03^{b}	0.50 ± 0.03^{a}	0.50 ± 0.03^{a}	0.27 ± 0.03^{b}	0.31 ± 0.03^{b}	*	*	*
C16:0	19.6 ± 0.14^{a}	20.9 ± 0.14^{b}	18.9 ± 0.16^{c}	19.3 ± 0.20^{a}	19.4 ± 0.20^{a}	20.5 ± 0.20^{b}	20.1 ± 0.20^{b}	*	*	*
C16:1	3.40 ± 0.10^{a}	3.39 ± 0.10^{a}	2.14 ± 0.10^{b}	2.94 ± 0.10^{ab}	2.75 ± 0.10^{a}	3.10 ± 0.10^{b}	3.10 ± 0.10^{b}	*	*	*
C18:0	9.60 ± 0.20^{a}	10.2 ± 0.20^{b}	12.1 ± 0.20^{c}	10.8 ± 0.20^{a}	10.8 ± 0.20^{a}	10.6 ± 0.20^{a}	10.4 ± 0.20^{a}	*	0.359	*
C18:1t	0.33 ± 0.01^{a}	0.33 ± 0.01^{a}	0.27 ± 0.01^{b}	0.30 ± 0.01^{ab}	0.30 ± 0.01^{a}	0.32 ± 0.01^{ab}	0.33± 0.01^{b}	*	*	*
C18:1c	29.4 ± 0.40^{a}	32.8 ± 0.40^{b}	26.0 ± 0.45^{c}	28.2 ± 0.45^{a}	29.5 ± 0.46^{bc}	29.2 ± 0.50^{ac}	30.7 ± 0.44^{b}	*	*	*
C18:2t	0.19 ± 0.01^{a}	0.21 ± 0.01^{a}	0.19 ± 0.01^{a}	0.13 ± 0.01^{a}	0.16 ± 0.01^{b}	0.24 ± 0.01^{c}	0.25 ± 0.01^{c}	0.952	*	*
C18:2c	24.0 ± 0.20^{a}	18.8 ± 0.20^{b}	23.7 ± 0.30a	22. 9± 0.30^{a}	21.6 ± 0.30^{b}	23.0 ± 0.30^{a}	21.2 ± 0.30^{b}	*	*	*
C18:3n6	0.23 ± 0.01^{a}	0.23 ± 0.01^{a}	0.19 ± 0.02^{b}	0.22 ± 0.02^{a}	0.21 ± 0.01^{a}	0.20 ± 0.02^{a}	0.24 ± 0.02^{a}	*	0.301	*
C18:3n3	0.98 ± 0.04^{a}	0.60 ± 0.04^{b}	0.80 ± 0.04^{c}	1.07 ± 0.04^{a}	0.96 ± 0.04^{a}	0.45 ± 0.05^{b}	0.67 ± 0.04^{c}	*	*	*

C20:1	0.58 ± 0.03^{a}	0.57 ± 0.03^{a}	0.59 ± 0.04^{a}	0.29 ± 0.04^{a}	0.36 ± 0.04^{a}	0.86 ± 0.04^{b}	0.83 ± 0.03^{b}	0.999	*	*
C:20:2n6	0.64 ± 0.02^{a}	0.45 ± 0.02^{b}	0.57 ± 0.02^{c}	0.58 ± 0.02^{a}	0.52 ± 0.02^{b}	0.58 ± 0.02^{a}	0.54 ± 0.02^{ab}	*	*	*
C:20:3n6	0.80 ± 0.02^{a}	0.82 ± 0.02^{a}	0.69 ± 0.03^{b}	0.84 ± 0.03^{a}	0.75 ± 0.03^{b}	0.75 ± 0.03^{b}	0.75 ± 0.03^{b}	*	*	*
C20:4n6	4.85 ± 0.20^{a}	5.36 ± 0.20^{a}	7.70 ± 0.20^{b}	6.41 ± 0.20^{a}	6.37 ± 0.20^{a}	5.40 ± 0.30^{b}	5.68 ± 0.20^{b}	*	*	*
EPA	0.36 ± 0.02^{a}	0.34 ± 0.02^{a}	0.26 ± 0.03^{b}	0.31 ± 0.03^{a}	0.33 ± 0.02^{a}	0.31 ± 0.03^{a}	0.33 ± 0.02^{a}	*	0.871	*
DHA	0.49 ± 0.04^{a}	0.66 ± 0.04^{b}	1.27 ± 0.04^{c}	0.89 ± 0.04^{a}	0.84 ± 0.04^{a}	0.70 ± 0.05^{b}	0.81 ± 0.04^{ab}	*	*	*
C24:0	1.30 ± 0.04^{a}	1.40 ± 0.04^{ab}	1.52 ± 0.04^{b}	1.53 ± 0.05^{a}	1.53 ± 0.05^{a}	1.24 ± 0.06^{b}	1.31 ± 0.05^{b}	*	*	*
C24:1	0.86 ± 0.04^{a}	0.82 ± 0.04^{a}	1.26 ± 0.04^{b}	1.02 ± 0.04^{a}	1.02 ± 0.04^{a}	0.86 ± 0.05^{b}	1.02 ± 0.04^{a}	*	*	*

Fatty acids (%) in chicken thigh (mean ± standard error). Different superscripts (a-c) indicate statistically significant differences at α=0.05 (p≤0.05) among groups of PS or S, according to the LSD criterion. *: p < 0.05; FA = Fatty acid; PS = Production system; S = Season; IS = Intensive system; ES = Extensive system; DPS = Dual- Purpose system; A= Autumn; W = Winter; Sp = Spring; Su = Summer; S × PS = interaction effect; C12:0 = lauric; C14:0 = myristic; C15:0 = pentadecanoic; C16:0 = palmitic; C16:1 = palmitoleic; C18:0 = stearic; C18:1t =trans-oleic; C18:1c = cis-oleic; C18:2t = trans- linoleic; C18:2c = cis- linoleic; C18:3n6 = gama linolenic; C18:3n3 = alpha linolenic; C20:1 = cis- gondoic; C20:2n6 = cis-eicosadienoic; C20:3n6 = dihomo- gama linolenic; C20:4n6 = arachidonic; EPA = eicosapentaenoic acid; DHA = docosahexaenoic acid; C24:0 = lignoceric; C24:1 = nervonic acid.

Table S5. Effect of PS and S on FA classes of chicken thigh (% of FA)

	PS			S				Significance (p-value)		
FA Type	IS	ES	DPS	A	W	Sp	Su	PS	S	S x PS
MUFA	34.9 ± 0.40^{a}	38.2 ± 0.40^{b}	30.5 ± 0.50^{c}	33.1 ± 0.50^{a}	34.2 ± 0.50^{ab}	34.6 ± 0.60^{b}	36.2 ± 0.50^{c}	*	*	*
PUFA	32.4 ± 0.30^{a}	27.3 ± 0.30^{b}	35.2 ± 0.40^{c}	33.2 ± 0.40^{a}	31.7 ± 0.40^{bc}	31.4 ± 0.50^{b}	30.4 ± 0.40^{b}	*	*	*
SFA	32.9 ± 0.20^{a}	34.7 ± 0.20^{b}	34.5 ± 0.25^{b}	34.0 ± 0.25^{a}	34.6 ± 0.25^{a}	34.2 ± 0.30^{a}	33.6 ± 0.25^{a}	*	0.243	*
UFA	67.4 ± 0.20^{a}	65.5 ± 0.20^{b}	65.7 ± 0.25^{b}	66.3 ± 0.24^{ab}	65.9 ± 0.24^{a}	66.0 ± 0.29^{ab}	66.1 ± 0.24^{b}	*	*	*
PUFA/SFA	0.99 ± 0.01^{a}	0.77 ± 0.01^{b}	1.03 ± 0.01^{a}	0.99 ± 0.01^{a}	0.93 ± 0.01^{b}	0.92 ± 0.01^{b}	0.91 ± 0.01^{b}	*	*	*
ω-3	1.76 ± 0.10^{a}	1.56 ± 0.10^{b}	2.27 ± 0.10^{c}	2.16 ± 0.10^{a}	2.07 ± 0.10^{a}	1.42 ± 0.10^{b}	1.81 ± 0.10^{c}	*	*	*
ω-6	30.7 ± 0.30^{a}	27.8 ± 0.30^{b}	32.9 ± 0.40^{c}	31.0 ± 0.40^{a}	29.6 ± 0.40^{b}	30.0 ± 0.40^{ab}	28.6 ± 0.40^{c}	*	*	*
ω-6/ω-3	20.9 ± 1.00^{a}	18.2 ± 1.00^{b}	17.6 ± 1.00^{b}	16.5 ± 1.00^{a}	15.7 ± 1.00^{a}	24.7 ± 1.00^{b}	18.6 ± 1.00^{c}	*	*	*
AI	0.38 ± 0.01^{a}	0.39 ± 0.01^{a}	0.35 ± 0.01^{b}	0.37 ± 0.01^{a}	0.37 ± 0.01^{ab}	0.39 ± 0.01^{b}	0.37 ± 0.01^{a}	*	*	*
TI	0.81 ± 0.01^{a}	0.88 ± 0.01^{b}	0.83 ± 0.01^{c}	0.81 ± 0.01a	0.82 ± 0.01^{ab}	0.88 ± 0.01^{c}	0.84 ± 0.01^{b}	*	*	*

FA class (%) in chicken thigh (mean ± standard error). Different superscripts (a-c) indicate statistically significant differences at α=0.05 (p≤0.05) among groups of PS or S, according to the LSD criterion. *: p < 0.05; FA = Fatty acid; PS = Production system; S = Season; IS = Intensive

system; ES = Extensive system; DPS = Dual- Purpose system; A= Autumn; W = Winter; Sp = Spring; Su = Summer; S × PS = interaction effect. MUFA = monounsaturated fatty acid; PUFA = polyunsaturated fatty acids; SFA = total saturated fatty acids; UFA = total unsaturated fatty acid; ω-3 = omega 3 fatty acids; ω-6 = omega 6 fatty acids; AI = atherogenicity index; TI = thrombogenicity index.

Table S6. Interaction effect of PS and S on thigh FA profile

	**A**			**W**			**Sp**			**Su**		
FA	IS	ES	DPS	IS	ES	DPS	IS	ES	DPS	IS	ES	DPS
C12:0	0.58 ± 0.10^{a}	0.79 ± 0.10^{a}	0.81 ± 0.10^{a}	0.82 ± 0.10^{a}	1.21 ± 0.10^{b}	1.17 ± 0.10^{b}	0.43 ± 0.10^{a}	0.55 ± 0.10^{a}	0.42 ± 0.10^{a}	0.40 ± 0.10^{a}	0.63 ± 0.10^{ab}	0.64 ± 0.10^{b}
C14:0	1.46 ± 0.10^{a}	1.39 ± 0.10^{a}	0.36 ± 0.10^{b}	1.34 ± 0.10^{a}	0.79 ± 0.10^{b}	0.95 ± 0.10^{b}	1.64 ± 0.10^{a}	0.93 ± 0.10^{b}	0.84 ± 0.10^{b}	1.03 ± 0.10^{a}	0.90 ± 0.10^{a}	0.95 ± 0.10^{a}
C15:0	0.45 ± 0.04^{a}	0.55 ± 0.04^{a}	0.44 ± 0.04^{a}	0.55 ± 0.04^{a}	0.53 ± 0.04^{a}	0.38 ± 0.04^{b}	0.24 ± 0.04^{a}	0.35 ± 0.04^{a}	0.23 ± 0.07^{a}	0.36 ± 0.04^{a}	0.27 ± 0.04^{a}	0.29 ± 0.04^{a}
C16:0	18.8 ± 0.30^{a}	20.6 ± 0.30^{b}	18.6 ± 0.30^{a}	18.8 ± 0.30^{a}	20.3 ± 0.30^{b}	19.1 ± 0.30^{a}	20.9 ± 0.30^{a}	21.0 ± 0.30^{a}	19.4 ± 0.40^{b}	20.0 ± 0.30^{a}	21.5 ± 0.30^{b}	18.8 ± 0.30^{c}
C16:1	3.10 ± 0.20^{a}	3.68 ± 0.20^{b}	2.06 ± 0.20^{c}	2.96 ± 0.20^{a}	3.14 ± 0.20^{a}	2.14 ± 0.20^{b}	3.96 ± 0.20^{a}	3.20 ± 0.20^{b}	2.10 ± 0.30^{c}	3.58 ± 0.20^{a}	3.54 ± 0.20^{a}	2.27 ± 0.20^{b}
C18:0	9.41 ± 0.40^{a}	10.3 ± 0.40^{a}	12.7 ± 0.40^{b}	10.0 ± 0.40^{a}	10.0 ± 0.40^{a}	12.4 ± 0.40^{b}	9.01 ± 0.40^{a}	10.4 ± 0.40^{b}	12.3 ± 0.50^{c}	9.92 ± 0.40^{a}	10.1 ± 0.40^{ab}	11.1 ± 0.40^{b}
C18:1t	0.33 ± 0.02^{a}	0.35 ± 0.02^{a}	0.22 ± 0.02^{b}	0.34 ± 0.02^{a}	0.29 ± 0.02^{b}	0.26 ± 0.02^{b}	0.30 ± 0.02^{a}	0.35 ± 0.02^{a}	0.31 ± 0.03^{a}	0.36 ± 0.02^{a}	0.36 ± 0.02^{a}	0.27 ± 0.02^{b}
C18:1c	29.9 ± 1.00^{a}	29.4 ± 1.00^{a}	25.1 ± 1.00^{b}	27.6 ± 1.00^{a}	34.7 ± 1.00^{b}	26.2 ± 1.00^{a}	30.7 ± 1.00^{a}	32.9 ± 1.00^{b}	24.0 ± 1.00^{c}	29.3 ± 1.00^{a}	34.2 ± 1.00^{b}	28.5 ± 1.00^{a}
C18:2t	0.11 ± 0.02^{a}	0.18 ± 0.02^{b}	0.11 ± 0.02^{a}	0.18 ± 0.02^{a}	0.17 ± 0.02^{a}	0.15 ± 0.02^{a}	0.20 ± 0.02^{a}	0.26 ± 0.02^{b}	0.26 ± 0.02^{b}	0.26 ± 0.02^{a}	0.23 ± 0.02^{a}	0.26 ± 0.02^{a}
C18:2c	24.9 ± 0.40^{a}	20.2 ± 0.40^{b}	23.6 ± 0.40^{a}	24.9 ± 0.40^{a}	18.0 ± 0.40^{b}	22.0 ± 0.40^{c}	23.2 ± 0.40^{a}	19.3 ± 0.40^{b}	26.4 ± 0.70^{c}	23.2 ± 0.40^{a}	17.7 ± 0.40^{b}	22.6 ± 0.40^{a}
C18:3n6	0.27 ± 0.03^{a}	0.25 ± 0.02^{a}	0.16 ± 0.03^{b}	0.25 ± 0.02^{a}	0.17 ± 0.02^{b}	0.21 ± 0.02^{ab}	0.17 ± 0.03^{a}	0.22 ± 0.04^{a}	0.20 ± 0.04^{a}	0.25 ± 0.02^{ab}	0.28 ± 0.03^{a}	0.19 ± 0.03^{b}
C18:3n3	1.10 ± 0.10^{ab}	0.96 ± 0.10^{a}	1.15 ± 0.10^{b}	1.66 ± 0.10^{a}	0.55 ± 0.10^{b}	0.67 ± 0.10^{b}	0.42 ± 0.10^{a}	0.43 ± 0.10^{a}	0.49 ± 0.10^{a}	0.76 ± 0.10^{a}	0.41 ± 0.10^{b}	0.85 ± 0.10^{a}
C20:1	0.38 ± 0.10^{a}	0.48 ± 0.10^{a}	0.20 ± 0.10^{b}	0.23 ± 0.10^{a}	0.48 ± 0.10^{b}	0.36 ± 0.10^{ab}	0.90 ± 0.10^{a}	0.66 ± 0.10^{b}	1.02 ± 0.10^{a}	0.83 ± 0.10^{a}	0.68 ± 0.10^{b}	0.97 ± 0.10^{a}
C:20:2n6	0.68 ± 0.03^{a}	0.53 ± 0.03^{b}	0.52 ± 0.03^{b}	0.67 ± 0.03^{a}	0.38 ± 0.03^{b}	0.50 ± 0.03^{c}	0.56 ± 0.03^{a}	0.48 ± 0.03^{a}	0.70 ± 0.05^{b}	0.67 ± 0.03^{a}	0.42 ± 0.03^{b}	0.55 ± 0.03^{c}
C:20:3n6	0.81 ± 0.05^{a}	0.99 ± 0.05^{b}	0.72 ± 0.05^{a}	0.82 ± 0.05^{a}	0.74 ± 0.05^{a}	0.71 ± 0.05^{a}	0.76 ± 0.05^{ab}	0.86 ± 0.05^{a}	0.62 ± 0.08^{b}	0.83 ± 0.05^{a}	0.68 ± 0.05^{b}	0.73 ± 0.05^{ab}
C20:4n6	4.82 ± 0.40^{a}	5.75 ± 0.40^{a}	8.67 ± 0.40^{b}	5.39 ± 0.40^{a}	5.36 ± 0.40^{a}	8.37 ± 0.40^{b}	4.11 ± 0.40^{a}	5.24 ± 0.40^{b}	6.86 ± 0.60^{c}	5.10 ± 0.40^{a}	5.10 ± 0.40^{a}	6.87 ± 0.40^{b}
EPA	0.35 ± 0.06^{a}	0.36 ± 0.03^{a}	0.22 ± 0.06^{b}	0.30 ± 0.04^{a}	0.35 ± 0.04^{a}	0.20 ± 0.05^{b}	0.29 ± 0.04^{a}	0.34 ± 0.04^{a}	0.28 ± 0.06^{a}	0.35 ± 0.04^{a}	0.31 ± 0.04^{a}	0.33 ± 0.04^{a}
DHA	0.45 ± 0.10^{a}	0.70 ± 0.10^{b}	1.51 ± 0.10^{c}	0.56 ± 0.10^{a}	0.67 ± 0.10^{a}	1.30 ± 0.10^{b}	0.37 ± 0.10^{a}	0.62 ± 0.10^{b}	1.10 ± 0.10^{c}	0.56 ± 0.10^{a}	0.65 ± 0.10^{a}	1.21 ± 0.10^{b}
C24:0	1.39 ± 0.10^{a}	1.52 ± 0.10^{ab}	1.68 ± 0.10^{b}	1.41 ± 0.10^{a}	1.40 ± 0.10^{a}	1.78 ± 0.10^{b}	1.04 ± 0.10^{a}	1.35 ± 0.10^{b}	1.34 ± 0.10^{ab}	1.38 ± 0.10^{a}	1.29 ± 0.10^{a}	1.26 ± 0.10^{a}
C24:1	0.74 ± 0.10^{a}	1.00 ± 0.10^{b}	1.30 ± 0.10^{c}	1.10 ± 0.10^{a}	0.78 ± 0.10^{b}	1.18 ± 0.10^{a}	0.69 ± 0.10^{a}	0.77 ± 0.10^{a}	1.12 ± 0.10^{b}	0.90 ± 0.10^{a}	0.74 ± 0.10^{a}	1.43 ± 0.10^{b}

Fatty acids (%) in chicken thigh (mean ± standard error). Different superscripts (a-c) indicate statistically significant differences at α=0.05 (p≤0.05) among groups of PS, according to the LSD criterion. *: $p < 0.05$; FA = Fatty acid; PS = Production system; S = Season; IS = Intensive system; ES = Extensive system; DPS = Dual- Purpose system; A= Autumn; W = Winter; Sp = Spring; Su =

Summer; C12:0 = lauric; C14:0 = myristic; C15:0 = pentadecanoic; C16:0 = palmitic; C16:1 = palmitoleic; C18:0 = stearic; C18:1t =trans-oleic; C18:1c = cis-oleic; C18:2t = trans- linoleic; C18:2c = cis- linoleic; C18:3n6 = gama linolenic; C18:3n3 = alpha linolenic; C20:1 = cis- gondoic; C20:2n6 = cis-eicosadienoic ; C20:3n6 = dihomo- gama linolenicC20:4n6 = arachidonic; EPA = eicosapentaenoic acid; DHA = docosahexaenoic acid; C24:0 = lignoceric; C24:1 = nervonic acid.

Table S7. Interaction effect of PS and S on thigh FA classes and nutritional indices

	**A**			**W**			**Sp**			**Su**		
FA class	IS	ES	DPS	IS	ES	DPS	IS	ES	DPS	IS	ES	DPS
MUFA	34.9 ± 1.00^{a}	35.3 ± 1.00^{a}	29.1 ± 1.00^{b}	32.6 ± 1.00^{a}	39.6 ± 1.00^{b}	30.4 ± 1.00^{a}	36.9 ± 1.00^{a}	38.2 ± 1.00^{a}	28.8 ± 1.00^{b}	35.2 ± 1.00^{a}	39.7 ± 1.00^{b}	33.7 ± 1.00^{a}
PUFA	33.2 ± 1.00^{a}	29.8 ± 1.00^{b}	36.5 ± 1.00^{c}	34.7 ± 1.00^{a}	26.2 ± 1.00^{b}	34.0 ± 1.00^{a}	29.9 ± 1.00^{a}	27.5 ± 1.00^{b}	36.8 ± 1.00^{c}	31.9 ± 1.00^{a}	25.7 ± 1.00^{b}	33.4 ± 1.00^{a}
SFA	32.1 ± 0.50^{a}	35.2 ± 0.50^{b}	34.6 ± 0.50^{b}	32.9 ± 0.50^{a}	34.3 ± 0.50^{b}	35.8 ± 0.50^{c}	33.3 ± 0.50^{a}	34.6 ± 0.50^{b}	34.6 ± 0.70^{b}	33.1 ± 0.50^{a}	34.8 ± 0.50^{b}	33.0 ± 0.50^{a}
UFA	68.1 ± 0.40^{a}	65.1 ± 0.40^{b}	65.6 ± 0.40^{b}	67.3 ± 0.40^{a}	65.9 ± 0.40^{b}	64.4 ± 0.40^{c}	66.8 ± 0.40^{a}	65.7 ± 0.40^{a}	65.7 ± 0.80^{a}	67.2 ± 0.40^{a}	65.4 ± 0.40^{b}	67.2 ± 0.40^{a}
ω-3	1.70 ± 0.10^{a}	2.01 ± 0.10^{a}	2.77 ± 0.10^{b}	2.63 ± 0.10^{a}	1.51 ± 0.10^{b}	2.08 ± 0.10^{c}	1.06 ± 0.10^{a}	1.33 ± 0.10^{a}	1.86 ± 0.20^{b}	1.70 ± 0.10^{a}	1.37 ± 0.10^{a}	2.40 ± 0.10^{c}
ω-6	31.5 ± 0.10^{a}	27.8 ± 0.10^{b}	33.7 ± 0.10^{c}	32.1 ± 0.10^{a}	24.8 ± 0.10^{b}	31.9 ± 0.10^{a}	28.8 ± 0.10^{a}	26.1 ± 0.10^{b}	35.0 ± 0.10^{c}	30.3 ± 0.10^{a}	24.3 ± 0.10^{b}	31.1 ± 0.10^{a}
ω-6.ω-3	22.8 ± 1.00^{a}	14.3 ± 1.00^{b}	12.5 ± 1.00^{b}	12.4 ± 1.00^{a}	18.7 ± 1.00^{b}	15.9 ± 1.00^{ab}	27.8 ± 1.00^{a}	21.3 ± 1.00^{b}	24.9 ± 2.00^{ab}	20.5 ± 1.00^{a}	18.5 ± 1.00^{a}	17.0 ± 1.00^{a}
PUFA. SFA	1.05 ± 0.02^{a}	0.85 ± 0.02^{b}	1.06 ± 0.02^{a}	1.07 ± 0.02^{a}	0.76 ± 0.02^{b}	0.95 ± 0.02^{c}	0.90 ± 0.02^{a}	0.79 ± 0.02^{b}	1.07 ± 0.03^{c}	0.97 ± 0.02^{a}	0.74 ± 0.02^{b}	1.02 ± 0.02^{a}
AI	0.37 ± 0.01^{a}	0.41 ± 0.01^{b}	0.32 ± 0.01^{c}	0.37 ± 0.01^{a}	0.37 ± 0.01^{a}	0.38 ± 0.01^{a}	0.42 ± 0.01^{a}	0.39 ± 0.01^{b}	0.36 ± 0.01^{b}	0.37 ± 0.01^{a}	0.40 ± 0.01^{b}	0.35 ± 0.01^{a}
TI	0.78 ± 0.01^{a}	0.86 ± 0.01^{b}	0.80 ± 0.01^{a}	0.75 ± 0.01^{a}	0.85 ± 0.01^{b}	0.87 ± 0.01^{b}	0.88 ± 0.01^{a}	0.90 ± 0.01^{a}	0.88 ± 0.02^{a}	0.82 ± 0.01^{a}	0.90 ± 0.01^{b}	0.78 ± 0.01^{a}

FA class (%) in chicken thigh (mean ± standard error). Different superscripts (a-c) indicate statistically significant differences at α=0.05 (p≤0.05) among groups of PS, according to the LSD criterion. *: $p < 0.05$; FA = Fatty acid; PS = Production system; S = Season; IS = Intensive system; ES = Extensive system; DPS = Dual- Purpose system; A= Autumn; W = Winter; Sp = Spring; Su = Summer; MUFA = monounsaturated fatty acid; PUFA = polyunsaturated fatty acids; SFA = total saturated fatty acids; UFA = total unsaturated fatty acid; ω-3 = omega 3 fatty acids; ω-6 = omega 6 fatty acids; AI = Atherogenicity index; TI = Thrombogenicity index.
